# Mechanical and Electrical Characterization of Piezoelectric Artificial Cochlear Device and Biocompatible Packaging

**DOI:** 10.3390/s150818851

**Published:** 2015-07-31

**Authors:** Youngdo Jung, Jun-Hyuk Kwak, Hanmi Kang, Wan Doo Kim, Shin Hur

**Affiliations:** Department of Nature-Inspired Nanoconvergence System, Korea Institute of Machinery and Materials, Daejeon 304-343, Korea; E-Mails: yjung@kimm.re.kr (Y.J.); jhkwak@kimm.re.kr (J.-H.K.); kanghanmi@kimm.re.kr (H.K.); wdkim@kimm.re.kr (W.D.K.)

**Keywords:** artificial cochlea, mechanical frequency separation, piezoelectric, biocompatible packaging

## Abstract

This paper presents the development of a piezoelectric artificial cochlea (PAC) device capable of analyzing vibratory signal inputs and converting them into electrical signal outputs without an external power source by mimicking the function of human cochlea within an audible frequency range. The PAC consists of an artificial basilar membrane (ABM) part and an implantable packaged part. The packaged part provides a liquid environment through which incoming vibrations are transmitted to the membrane part. The membrane part responds to the transmitted signal, and the local area of the ABM part vibrates differently depending on its local resonant frequency. The membrane was designed to have a logarithmically varying width from 0.97 mm to 8.0 mm along the 28 mm length. By incorporating a micro-actuator in an experimental platform for the package part that mimics the function of a stapes bone in the middle ear, we created a similar experimental environment to cochlea where the human basilar membrane vibrates. The mechanical and electrical responses of fabricated PAC were measured with a laser Doppler vibrometer and a data acquisition system, and were compared with simulation results. Finally, the fabricated PAC in a biocompatible package was developed and its mechanical and electrical characteristics were measured. The experimental results shows successful frequency separation of incoming mechanical signal from micro-actuator into frequency bandwidth within the 0.4 kHz–5 kHz range.

## 1. Introduction

Human ear is a miniaturized acoustic transducer of great sensitivity (20 μPa–60 Pa) and wide dynamic frequency range (20 Hz–20 kHz). Human cochlea, a snail shell-shaped organ in the inner ear, plays an important role in hearing [1]. Patients with damaged cochlea require artificial cochlear implants to restore their hearing. Conventional artificial cochlear implants suffer from high power consumption and exposure of hearing loss due to their external units [2]. In an effort to overcome the shortcomings of conventional artificial cochlear implants, there have been many studies to mimic the function of a basilar membrane in human cochlea and develop the artificial cochlea [3,4,5,6,7,8,9]. However, the focus of research was to mimic the frequency separation of a basilar membrane and did not show signal conversion of mechanical movements of the basilar membrane to electrical signals. Recently, signal conversion of a basilar membrane upon sound signal input was reported over a narrow frequency bandwidth [10,11]. Previously in our group, we reported the development and characterization of an artificial basilar membrane made of piezoelectric polymer capable of frequency separation and electric signal generation within human hearing range. However, while the frequency range of human voice conversation is below 2 kHz, the frequency separation range of the previously reported device without a liquid chamber was 2.5 kHz–13.5 kHz [12].

In this paper, we report the development of a piezoelectric artificial cochlea (PAC) capable of frequency separation over 450 Hz–5000 Hz. The piezoelectric artificial membrane was assembled with a liquid chamber and the frequency separation behaviors and electrical signal measurement were carried out with a micro-actuator attached to the liquid chamber to analyze the function of PAC as a sound frequency analyzer. The experimental results were analyzed and compared with simulation results. Furthermore, an integrated PAC with PCB substrate having multiple electrical connections and implantable packages was developed, and its mechanical and electrical characteristics were measured. 

## 2. Design and Simulation

**Figure 1:**
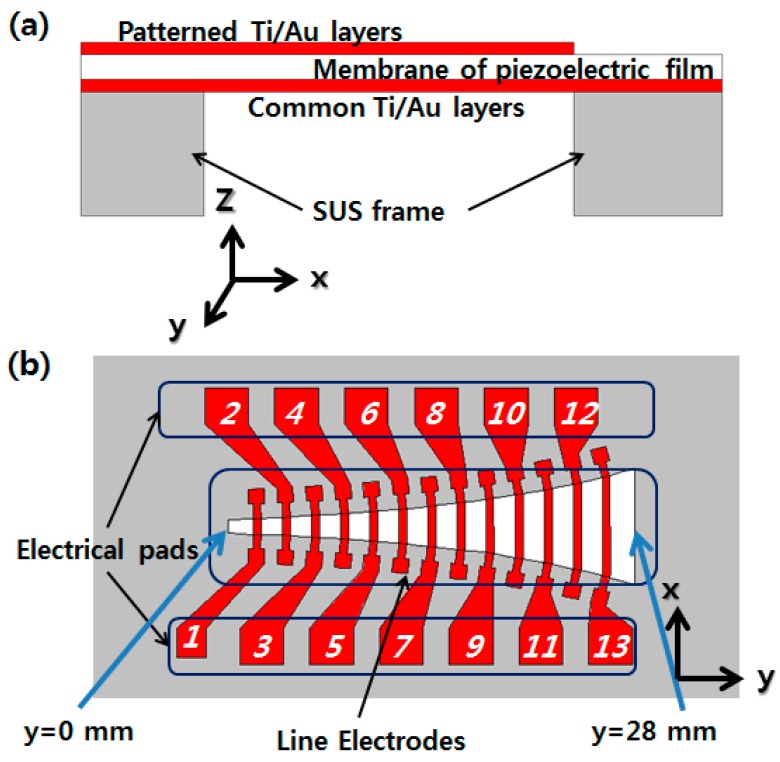
2D schematic of membrane part design of PAC: from side view (**a**); from top view (**b**).

**Figure 2:**
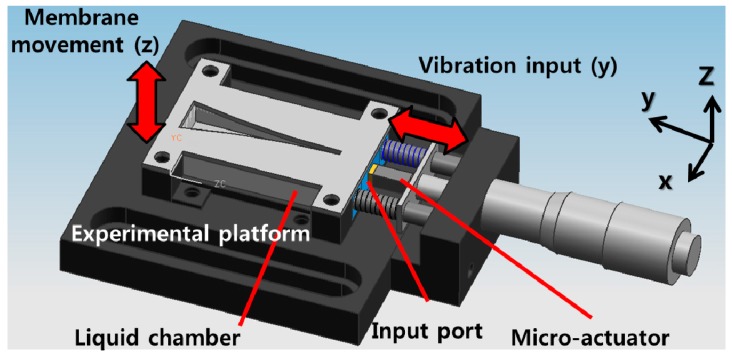
3D drawing of packaged part of PAC with SUS frame of membrane part placed over the liquid chamber.

The PAC consists of an artificial basilar membrane part and a packaged part. The membrane part has a piezoelectric polymer film as a membrane material, a stainless use steel (SUS) frame defining the shape of the membrane. On the top of the membrane film, there are patterned electrical lines and pads, while on the bottom, there is a common electrical layer (Figure 1). The packaged part consists of a liquid chamber with an input port and the membrane part assembled with the packaged part is tested on a packaged platform with a micro-actuator attached (Figure 2).

The membrane part is assembled on the liquid chamber and the liquid chamber is fixed onto the experimental platform, which can be bolted securely to a vibration isolation table. A micrometer assembled with the experimental platform can adjust the position of the micro-actuator precisely such that the tip of the micro-actuator is placed precisely on the surface of the input port. The dimension of liquid chamber is 10 mm × 37 mm × 5 mm and the tip area of micro-actuator on the input port is 3 mm × 2 mm.

The length in y axis of an opening in the SUS frame is 28 mm and the width in *x* axis varies logarithmically from 0.97 mm to 8.0 mm. The piezoelectric polymer film is made of 25.4 μm thick polyvinylidene difluoride (PVDF) film. A thin metal layer covers the entire bottom side of piezoelectric polymer film as a common electrical pad, and 13 line electrodes across the membrane with 13 electrical pads are designed on the top side (Figure 1). The width (along the *y*-axis) of each line electrode is 0.5 mm and the length (along the *x*-axis) is the same as the width of the membrane at each position. The line electrodes are placed over the entire width of the membrane to make the orthotropic membrane like a human basilar membrane in order to improve the membrane response. The spacing between adjacent line electrodes is 1.5 mm.

**Figure 3:**
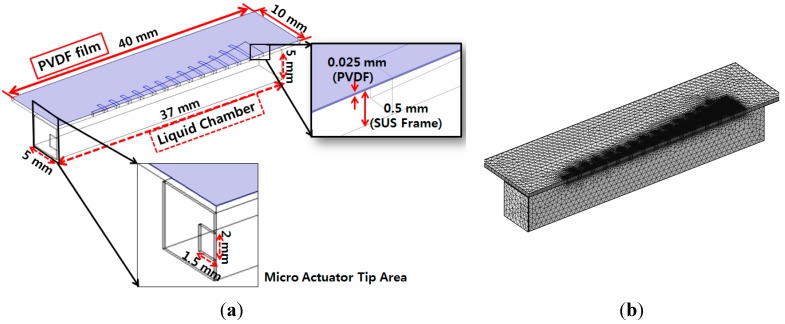
Finite element model of designed piezoelectric artificial cochlea: half symmetric model with its geometry and dimensions (**a**); half symmetric meshed model (**b**).

Multiphysics finite element analysis (FEA) was carried out using COMSOL Multiphysics^®^ software (COMSOL, Inc., Palo Alto, CA, USA) to simulate the mechanical behaviors and electrical signal generation of the designed PAC. Figure 3a shows the geometry of the finite element model and Figure 3b shows the half symmetric meshed model.

**Figure 4:**
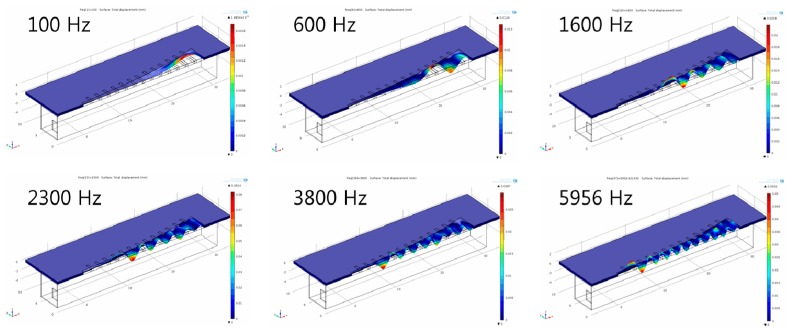
Frequency separation analysis with finite element model of the designed PAC with a liquid chamber: The membrane deformation indicates relative local resonant displacement of the membrane. Position of maximum resonant displacement (MRD) at each local resonant frequency (LRF) moves closer to base area at higher frequency (graphs not drawn to scale).

The finite element model was constructed to be half of the membrane part and liquid chamber, and symmetric boundary conditions were applied to reconstruct the whole design simulation. The mode frequencies and position of maximum resonant displacement (MRD) at each mode frequency was analyzed first with the finite element model without a liquid chamber. The position of MRD moves gradually from apex area (wide width) at lowest model frequency to base (narrow width) area at higher mode frequency. The lowest mode frequency was 1.44 kHz and the position of MRD was 24.4 mm from the base (y = 0 mm), while the position of MRD was 16.5 mm from the base at the mode frequency of 4.42 kHz. The FEA for frequency separation of the PAC included the simulation of the input vibration loads of various frequencies applied on the input port of the liquid chamber, fluid–structure interaction (FSI) between the input port and the fluid in the liquid chamber, and FSI between the fluid and the piezoelectric membrane. Sinusoidal displacement load modeling the movement of the micro-actuator tip was applied on the movable area of the input port. The frequencies of the applied loads ranged from 0.1 kHz–20 kHz. Figure 4 shows the deformation of the membrane of the finite element model with a liquid chamber at different frequencies. Similarly to those of modal analysis, the position of maximum displacement (peak with red color) moves gradually from apex area (wide width) to base (narrow width) area as the frequency of applied loads increases. 

**Figure 5:**
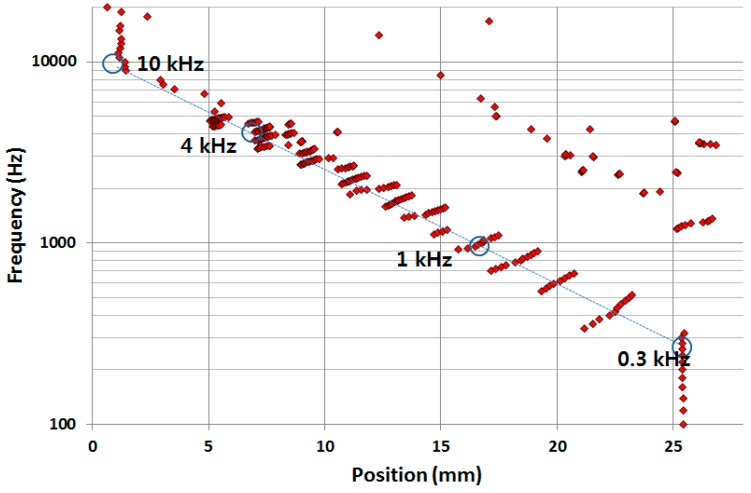
Frequency separation FEA of the designed PAC with a liquid chamber: each red dot corresponds to the position having maximum displacement at each frequency.

Figure 5 show the simulation results of the frequency separation properties of the designed PAC with a liquid chamber. The results demonstrated that the designed PAC responded tonotopically to incoming signals of varying frequencies from 0.3 kHz near the apex area and up to 10 kHz near the base area in a similar fashion as a human basilar membrane. The dots on the right upper side can be regarded as signal noise attributed to the reflected waves near the apex or a higher mode in the transverse direction. 

The first five modal frequencies (F_M_) from modal analyses without a liquid chamber and the first five local resonant frequencies (F_R_) from the FEA for frequency separation of the PAC with a liquid chamber are listed in Table 1 with the positions of MRD (L_M_ for mode analysis, L_R_ for frequency separation simulation). The L_M_ and L_R_ values are quite similar, but the local resonant frequencies in liquid were reduced four times compared to those of modal frequencies due to mass loading effect on the membrane. In order to lower the frequency response range of PAC, one should modify a PAC with larger membrane area on the design perspective and/or fabricate a membrane part with more flexible materials. However, there is limitation in increasing its dimension for a PAC to be implanted in the human body. However, this mass loading effect enables the designed PAC to cover the lower part of audible frequency range without increasing the size of the overall PAC.

**Table 1:** FEA comparison between modal frequencies (F_M_) and the position of MRD (L_M_) at each F_M_ of the membrane part without a liquid chamber and the local resonant frequencies (F_R_) and position of MRD (L_R_) at each F_R_ of whole PAC with a liquid chamber.

Mode Analysis without a Liquid Chamber		FSI Analysis with a Liquid Chamber		Frequency Ratio
F_M_ (kHz)	L_M_ (mm)	F_R_ (kHz)	L_R_ (mm)	F_M_/F_R_
1.44	24.4	0.320	25.5	4.5
2.13	21.3	0.460	22.7	4.63
2.85	19.3	0.640	20.3	4.45
3.60	17.6	0.820	18.5	4.39
4.42	16.5	1.06	17.2	4.17

## 3. Fabrication and Experiments

The fabrication process involves a microfabrication process to form patterned line electrodes and electrical pads, a corona poling process to enhance the piezoelectricity of piezoelectric polymer film, and an assembly process of a membrane part and a packaged part. The piezoelectric polymer film used was PVDF film of thickness 25.4 μm (Kynar^®^ Film, Professional Plastics, Singapore). 20 nm/200 nm thick titanium (Ti)/gold (Au) layers were deposited with a shadow mask on the glossy side of PVDF film with e-beam evaporator. The shadow mask was made of stainless steel and has openings for 13 patterned line electrodes and electrical pads. Thus, with one deposition process without photolithography, electrical patterns were realized on the PVDF film. Additional Ti/Au layers were deposited on the opaque side as a common electrical pad for harvesting piezoelectric signal. 

**Figure 6:**
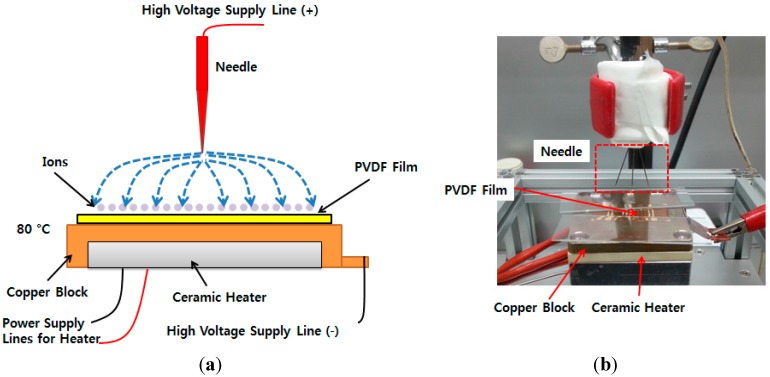
Schematic diagram of corona poling device (**a**); setup of corona poling device (**b**).

A corona poling process was conducted on the processed PVDF film to improve its piezoelectricity at 80 °C for 20 min with 6.5 kV (Figure 6). The piezoelectric constant of the poled piezoelectric film was measured with PiezoMeter System (PM300, Piezotest, UK). The d33 value of piezoelectric constant of the corona poled PVDF film was was around 3.5 pC/N.

**Figure 7:**
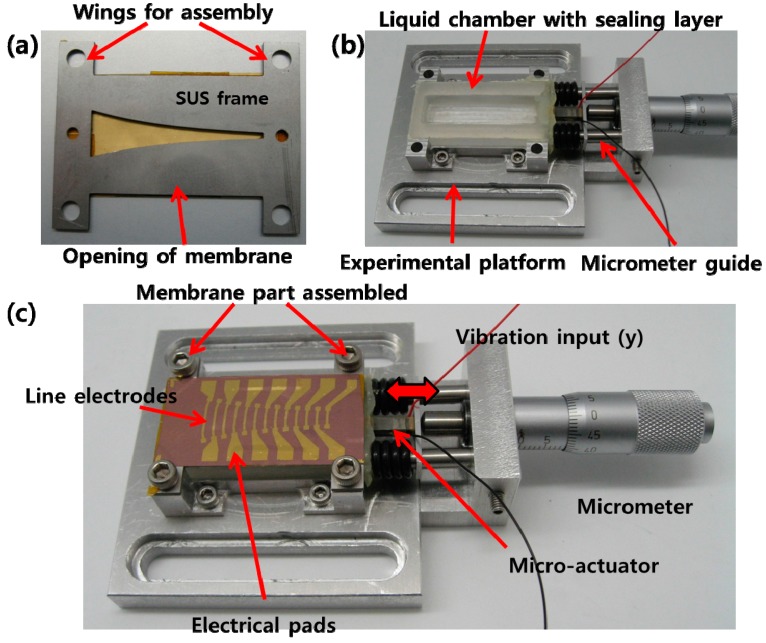
Photographs of bottom side of membrane part (**a**); packaged part (**b**); assembled PAC with liquid chamber on the experimental platform (**c**).

An SUS frame having openings that defines the membrane shape and fluid introduction holes and wings for secure assembly on the packaged part was prepared with precision machining of line saw process. The processed PVDF film was attached on the SUS frame constituting a membrane part. An experimental platform and a liquid chamber were prepared with conventional machining process. The micro-actuator used was PICMA^®^ Stack Multilayer Piezo actuator (P-883.11, Physik Instruments (PI) Ceramic, Lederhose, Germany) and assembled on the experimental platform with a micrometer. The fabricated PAC is shown in Figure 7. The membrane part, liquid chamber, and micro-actuator with micrometer are assembled firmly to the experimental platform with bolts.

**Figure 8:**
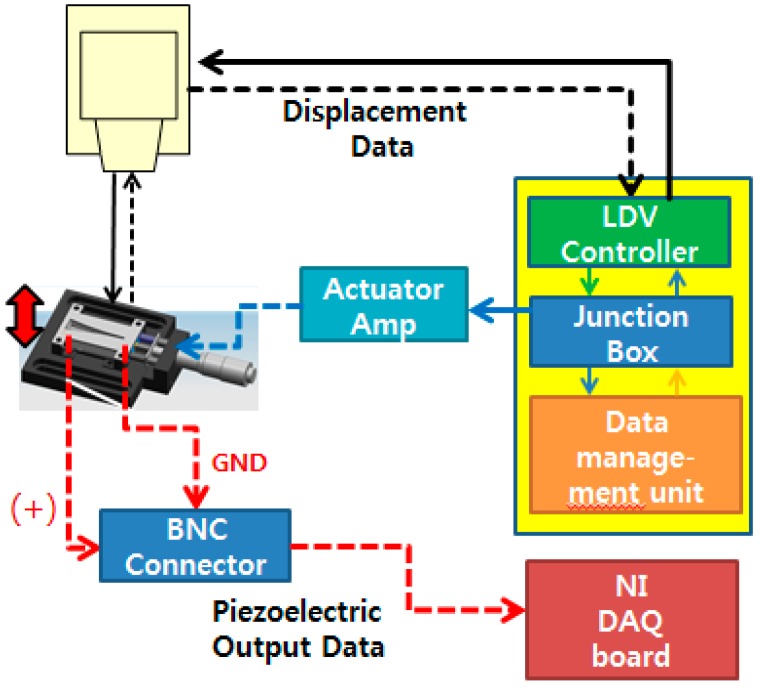
Schematic of experimental setup for characterization of the developed PAC: solid lines are connections for control and actuation signal and dotted lines are connections for measuring vibration and piezoelectric signals.

The overall experimental setup to characterize the fabricated PAC is shown in Figure 8. The fabricated PAC was characterized mechanically and electrically by measuring its vibratory behaviours and electrical output signals upon vibratory input from the micro-actuator. The membrane vibration or displacement in z direction was measured with a scanning laser Doppler vibrometer system (PSV-I-400 LR and OFV-505, Polytec, Waldronn, Germany) and the electrical output signals from the electrical pads on the membrane were acquired with a high accuracy data acquisition (DAQ) module (NI Pxle-4497, National Instruments, Austin, TX, USA). The electrical signals from a function generator in the junction box of the LDV system controlled the magnitude and frequency of vibratory inputs from the micro-actuator. The input signal applied to the actuator amplifier was in the form of white noise (20 Hz–20 kHz, 10 Vpp).

**Figure 9:**
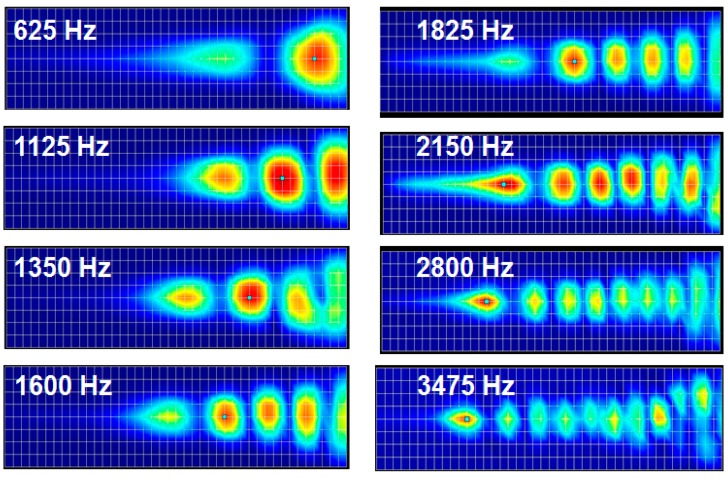
2D displacement measurement of membrane movement by LDV: The apex is near the right end of image and the base is near the left end of image.

The frequency response of the micro-actuator operated by the actuator amplifier showed that it has near flat frequency response with 3 dB cut off frequency around 6 kHz while there was a slight fluctuation near 4 kHz. The liquid chamber of the PAC was completely filled with de-ionized water without leaving any air bubble through the fluid introduction holes. Trapped air bubbles disrupt the wave propagation from the input port to the membrane and distort the frequency response of the PAC. The prepared PAC was bolted securely to an active vibration isolation table to minimize the effects of any unwanted external vibration. Figure 9 shows the frequency separation properties of the developed PAC over the whole membrane upon the mechanical vibration input from the micro-actuator. The position of maximum displacement moved gradually from apex (with wide width) to base (with narrow width) as the frequencies of input signals increased.

**Figure 10:**
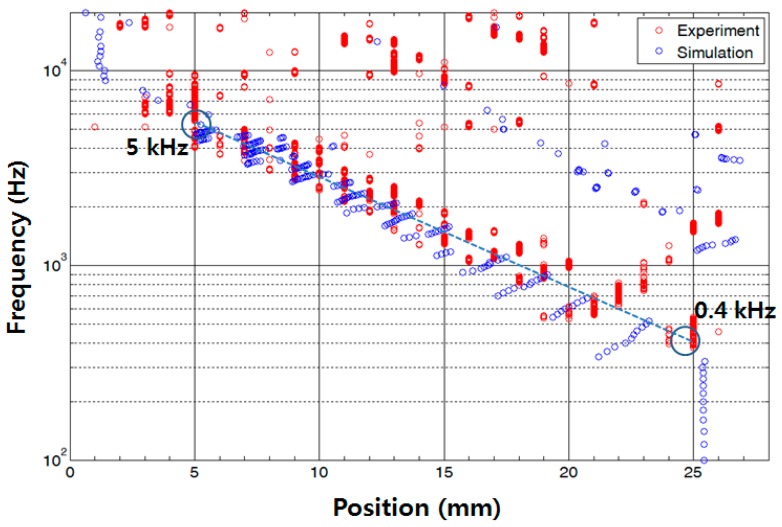
Frequency separation analysis of the designed PAC: each red (experiment) and blue (simulation) dot corresponds to the position having maximum displacement at each frequency expressed by the distance from the base.

To further investigate the vibratory behaviours of the membrane, the position of maximum displacement at each frequency analysed from the measurement data (red dots) was plotted with those from the FEA (blue dots). The experimental frequency separation properties of the developed PAC above 5 kHz were not as clear as those from the FEA simulation results partly due to the limitation of frequency response of the micro-actuator. However, the experimental results of the frequency selectivity of the designed PAC matched well with the simulation results as the designed PAC responded tonotopically to incoming signals of varying frequencies from 0.4 kHz near the apex area and up to 5 kHz near the base area (Figure 10). There were also dots positioned upper right corner of the straight line between (25 mm, 0.4 kHz) and (5 mm, 5 kHz) due to the effect of the reflected wave near the apex or higher mode in the transverse direction as expected in the FEA. 

**Figure 11:**
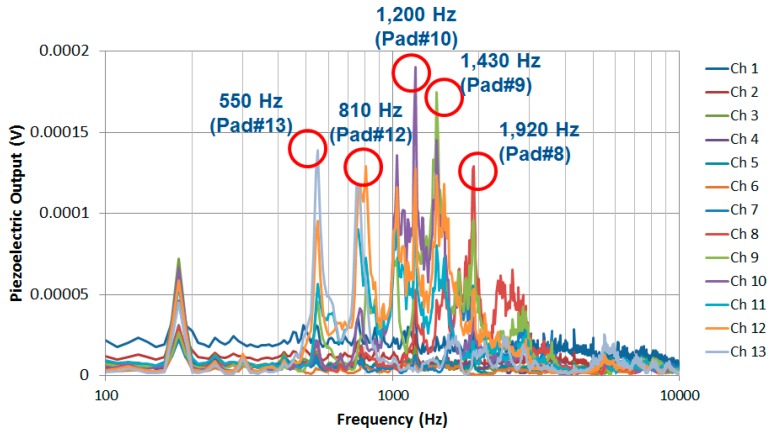
Electric signal outputs of the designed PAC.

The electric signals of the PAC generated on each line electrode at various frequencies were measured at each electrical pad. The electrical pad that generated largest electric signal output was identified at each frequency. The electrical pad #13 (near apex) generated maximum piezoelectric signal of 0.14 mV at the lower frequency band around 0.5 kHz and the electrical pad #10 and #8 responded most at the frequency band around 1.2 kHz and 1.9 kHz, respectively (Figure 11). The electrical pad #10 generated the maximum piezoelectric signal of 0.19 mV at 1.2 kHz. The magnitude of micro-actuator vibration at 1.2 kHz was 18 nm, while human tympani membrane displacements were around 100 nm with a sound put of 94 dBSPL [13]. However, from electrical pad #1–#7, the piezoelectric signal outputs measured were low and could not be analysed further as the magnitude of membrane vibration is much smaller near the base compared to those near the apex. The signal peak at 180 Hz was from the third harmonic of 60 Hz noise signals during the experiments. The output signal level was relatively low considering the third harmonic of 60 Hz noise signal, but was sufficient as an input signal to operate the current stimulator under development for stimulating the hearing nerve.

**Figure 12:**
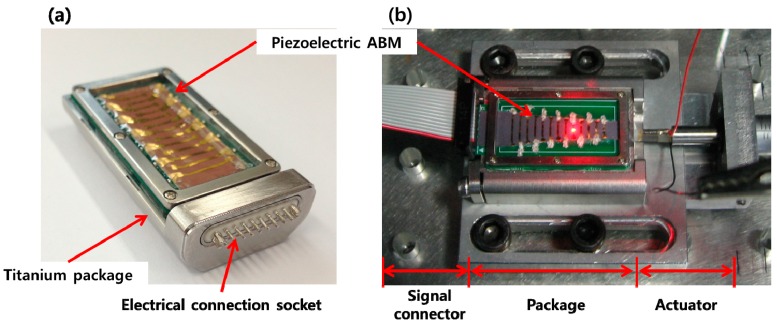
Fabrication and test setup result of the PAC: the fabricated biocompatible PAC (**a**); test setup of PAC device (**b**).

**Figure 13:**
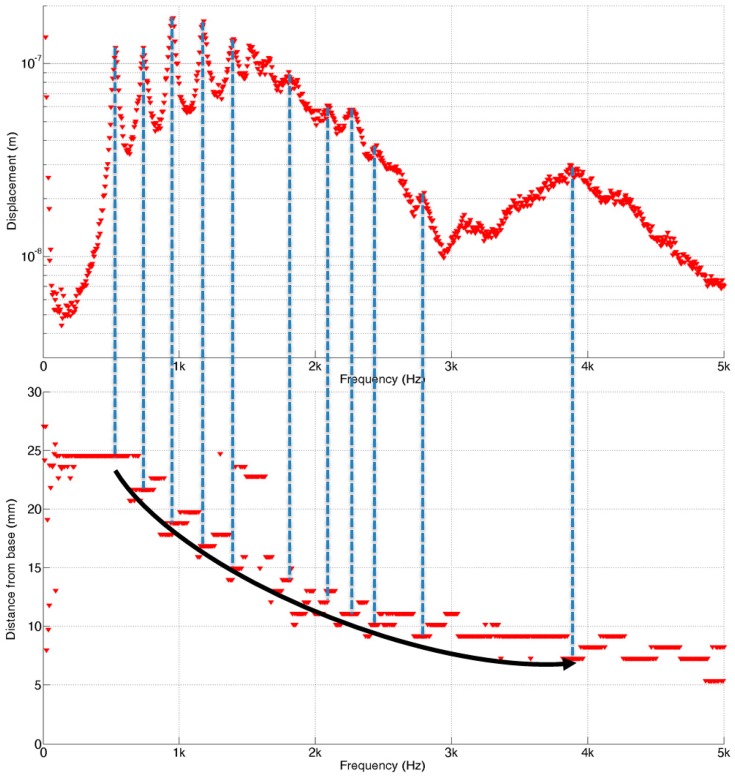
Frequency separation properties of the packaged PAC: top graph represents the maximum displacement on the membrane and bottom graph represents the position having maximum displacement at each frequency expressed by the distance from the base.

**Figure 14:**
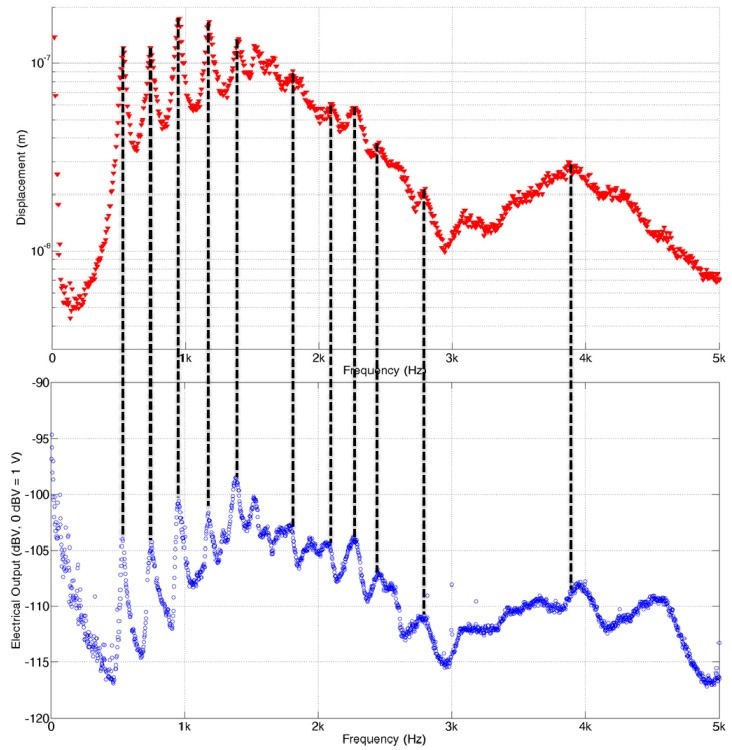
Vibratory and electrical characteristics of the packaged PAC: top graph represents the maximum displacement on the membrane and bottom graph represents the maximum electrical output from 13 electrical pads.

In order for a PAC to be implantable in the human body, both the packaged part and membrane part was modified and refabricated to be more compact and biocompatible. The packaged part was made with titanium, and liquid sealing layers were fabricated with medical grade silicone rubber. Also, the FEA results were considered to redesign the liquid chamber to have an artificial round window made of thin silicone rubber film near the apex to reduce the signal noise arising from wave reflection on the wall of the liquid chamber near apex. The membrane part was constructed on a printed circuit board (PCB) instead of a SUS frame for a more compact electrical connection. The electrodes on the membrane were electrically connected to the PCB by silver paste and the PCB was connected to the external electrical wires through the embedded multiple electrical connector. The embedded multiple electrical connector can be plugged in by an external multiple electrical connection socket for simple connection and disconnection of the signal outputs from the membrane part. Fabrication result of the biocompatible PAC device and test setup for the PAC device is shown in Figure 12. The mechanical and electrical characteristics of the redesigned PAC were measured and demonstrated its frequency separation capability over the human voice frequency range (Figure 13). Below 1 kHz, three distinct local resonant frequencies were observed both in their vibratory and electrical behaviours (Figure 14). 

## 4. Conclusions

In this study, we have presented the development of a piezoelectric artificial cochlea mimicking the function of human cochlea. The PAC had a packaged part that provides artificial stapes, oval window and cochlear duct for frequency separation. The characteristics of the PAC were simulated by the FEA of mode analysis without a liquid chamber, and frequency separation with a liquid chamber, and were compared with the measurement results from its vibratory behaviour and electrical signal generation. The human audible hearing range is 20 Hz–20 kHz, but more importantly, the voice band is 0.3 kHz–3.5 kHz. The highest note reproducible by the average female human voice is C6 of 1046.5 Hz. The FEA of frequency selectivity demonstrated that the designed PAC has frequency selectivity from 0.3 kHz–10 kHz. The PAC was fabricated with simple microfabrication and precision micromachining processes along with corona poling of piezoelectric polymer film. The fabricated PAC was characterized by measuring its vibratory behaviour with a scanning LDV system. The experimental results matched well with the simulation results and showed the frequency selectivity of the PAC over 0.4 kHz–5 kHz which covers most of the voice band. As the frequency went higher, the local resonant position moved gradually from the apex area toward base area. The electrical signals measured showed the successful frequency separation of the PAC into five frequency bands below 2 kHz, but due to the experimental setup issues and non-uniform piezoelectricity over the membrane, the electrical signals could not be measured near the base area. The maximum electrical signal was 0.19 mV at 1.2 kHz from the electrical pad #10. The packaged PAC device made of biocompatible material shows more clear frequency separation characteristics than the unpackaged PAC device without higher mode components.

Compared to the typical piezoelectric constant of poled PVDF film, the measured values were quite low. More elaborate poling processes, such as stretching during corona poling, would enhance its piezoelectricity and, eventually, the output signal levels. While the electrodes were designed such that they can make the membrane orthotropic, the design could reduce the output signal level since the piezoelectric outputs near the edge and the central area of the membrane have opposite polarity as the membrane vibrates. Electrode design modification such as narrower electrode near the edge and fabrication process change such as using different types of materials for the edge area and central area can further enhance the output signal levels. To improve the performance of the PAC and resolve the issues discussed earlier, aspects of the performance improvement such as electrical noise suppression, membrane tension control, device miniaturization and an implantable technique will be investigated further. The presented PAC is another step towards the development of total implantable cochlear systems requiring much less power and exhibiting more natural performance in sound conversion compared to conventional cochlear implants, and thus, contributing to an enhancement in the quality of life of those suffering from hearing loss.
